# Psychological profile of patients with Brugada syndrome and the impact of its diagnosis and management

**DOI:** 10.1038/s44325-024-00042-6

**Published:** 2025-01-13

**Authors:** Francesco Santoro, Luigi Di Biase, Antonio Curcio, Alessandra Margaglione, Massimo Tritto, Enrico Baldi, Ilaria Ragnatela, Matteo Anselmino, Gerardo Nigro, Ivana Leccisotti, Mario Altamura, Antonello Bellomo, Natale Daniele Brunetti, Antonio Rapacciuolo

**Affiliations:** 1 Department of Medical and Surgery Sciences, Cardiology Unit, University of Foggia, Foggia, Italy; 2 Department of Cardiology, Montefiore Medical Center, Bronx, NY USA; 3 Division of Cardiology, Department of Pharmacy, Health Sciences and Nutritional Sciences, University of Calabria, Rende, Italy; 4 Electrophysiology and Cardiac Pacing Unit, IRCCS Humanitas Research Hospital, Rozzano, Italy; 5 Division of Cardiology, Fondazione IRCCS Policlinico San Matteo, Pavia, Italy; 6 Division of Cardiology, Cardiovascular and Thoracic Department, “Città della Salute e della Scienza” Hospital and Department of Medical Sciences, University of Turin, Turin, Italy; 7 Cardiology Unit, Department of Medical Translational Sciences, University of Campania “Luigi Vanvitelli”—Monaldi Hospital, Naples, Italy; 8 Department of Clinical and Experimental Medicine, Psychiatry Unit, University of Foggia, Foggia, Italy; 9 Division of Cardiology, Department of Advanced Biomedical Sciences, Federico II University, Naples, Italy

**Keywords:** Cardiology, Cardiovascular diseases

## Abstract

Brugada syndrome (BrS) is an arrhythmic hereditary disorder affecting mainly males, aged 30–50 years. Type D personality has a prevalence of 32.7% among BrS patients and 15% of these patients have an history of psychiatric disorders. One out of six BrS patients could develop anxiety/depression after BrS diagnosis or after the implantation of a defibrillator. This review evaluates the psychological profile of BrS patients, the impact of its diagnosis, and potential tools to evaluate these features.

## Introduction

Brugada syndrome (BrS) is a hereditary disorder, with autosomal dominant transmission and incomplete penetrance, manifesting mainly in males aged 30–50 years with resting syncope, nocturnal agonal breathing, major ventricular arrhythmias, and sudden cardiac death^1^. The diagnosis of BrS has significant implications for clinical management and genetic counseling. BrS patients often face crucial decisions, including the choice to accept drug therapies or procedural interventions, as well as genetic implications for family members^2,3^.

The diagnosis of BrS can be made by ECG findings of a type 1 BrS pattern and other clinical features, such as documented polymorphic ventricular tachycardia (PVT)/Ventricular fibrillation (VF), arrhythmic syncope, or family history of BrS^1^. BrS Type 1 ECG pattern consists of a J point elevation of >2 mV with coved ST elevation and T wave inversion in at least one right precordial ECG lead, V1 or V2, located at the second, third, or fourth intercostal spaces^1^. This pattern may be found spontaneously or could be induced during fever or due to sodium channel blocking drugs exposure. If such a pattern is found, it is necessary to exclude other underlying conditions^1^.

Other BrS ECG patterns are type 2 (saddle-back pattern) and type 3 that are less well defined (right precordial ST elevation < 1 mm saddle-back, coved type, or both). These patterns are not diagnostic “per se” but in selected cases could be evaluated with sodium channel blocking drugs test.

The diagnostic-therapeutic course following a diagnosis of BrS is complex and involves taking measures such as educating the patient to avoid states of hyperpyrexia or excessive alcohol intake, as well as a list of sodium channel blocking drugs and substances to avoid^4^. In-depth diagnostics such as testing with sodium-channel blocking drugs may be necessary, up to the possibility of an electrophysiological study with programmed electrical stimulation or the implantation of a loop-recorder^5,6^.

Proband genetic diagnostic tests and screening of family members become necessary once the diagnosis of BrS is made. This is often difficult and generates concern in patients and their family. The first therapeutic approach will be guided by the presentation setting. Generally, there is an acute pharmacological treatment only in case of arrhythmic storm, that requires therapy with endo-venous Isoproterenol^7^.

In BrS patients at highest risk of sudden cardiac death, the long-term therapy that has shown the best efficacy is ICD implantation^7^.

Many variables can affect illness perceptions of patients diagnosed with inherited cardiac diseases. First, the type of disease and its implications in terms of prognosis and treatment^8^. Secondly, patient-related factors, including his or her psycho-aptitude profile, the family, socio-economic and cultural context, comorbidities (often involving polypharmacotherapy), and the availability of a caregiver^9^.

These factors may thereby influence crucial aspects of BrS management^10^ as well as patients’ quality of life.

Focusing on these aspects may help in a mindful framing of these patients. This may lead to a more complete follow-up, admitting the possibility of psychological evaluation and counseling from the earliest stages of diagnosis. Aim of the review is to evaluate current literature on psychological profile of BrS patients, the impact of its diagnosis and potential tools to evaluate these features.

## Methods

We identified relevant English-language publications through a PubMed search using the keywords “Brugada Syndrome”, in March 2024 in different combinations with: anxiety, depression, psychological impact, psychological disorders. We completed this search by cross-referencing published articles and also performed a hand search of major journals. We have restricted the citations to the most relevant and informative publications.

One hundred and twenty studies were identified through database search and 36 duplicates were removed. Seventy-five unique records were screened and 44 of them were excluded. Thirty-one full-text articles were assessed for eligibility and four full articles were selected for this review (Table 1).

**Table 1:** List of papers detailing the clinical features and psychological profile of patients with Brugada syndrome (BrS)

*Study*	year	Num. of patients	Mean age	Male (%)	History of Psychiatric Disorders Before BrS diagnosis	new-onset depression or anxiety after BrS diagnosis
Jespersen et al. ^19a^	2023	263	46	76%	40 (15.2%)	15.7%; *n* = 35
*symptomatic*		101	46.1	77.2%	0	19.3%
*asymptomatic*		122	45.8	84%	0	13%
Six et al. ^3^	2023	162	49	59%	NA	28.4% (mental disorders) 32.7% (Type D personality)
Sutjaporn et al. ^18^	2022	29	45	96%	NA	17.2% (anxiety) 13.9% (depression)
Probst et al. ^8^	2011	190	53	79%	NA	49% anxiety on health status (41% moderate, 8% severe);

^a^In this study patients with history of psychiatric disorders before BrS diagnosis were excluded, only patients with new onset of anxiety and depression after BrS diagnosis were included.

### Diagnosis of Brugada syndrome and potential psychological consequences

When evaluating the quality of life of patients diagnosed with BrS, one must consider the setting where the diagnosis occurs. Sometimes, BrS diagnosis is made during genetic screening after an acute event often occurred to a first-degree relative, such as a resuscitated cardiac arrest or sudden death^9^. In most instances the diagnosis is incidental during a routine ECG recorded for different reasons in subjects considering themselves definitely healthy. Obviously, these settings generate acute distress^10,11,12,13^ whose duration and intensity are higher in patients who experienced the unexpected death of a relative^14^.

Mental distress, anxiety and doubts about the need for medical treatment are well known factors associated with reduced drug adherence^15^, as well as with higher morbidity and mortality^16,17^. In a single center study from Taiwan including 29 highly symptomatic BrS patients there were increased levels of anxiety and depression. As a matter of fact, there was a prevalence of 17.2% and 13.9% of anxiety or depression, respectively^18^. In a recent Danish study^19^, it was found that approximately one out of six (16%) BrS patients developed anxiety/depression after diagnosis. Moreover, when only symptomatic patients were considered at diagnosis (resuscitated arrest; occurrence of ventricular tachycardia; syncope), the incidence of new-onset depression/anxiety was higher^19^ (Table 1).

### Which tools can be used to evaluate the level of well-being of BrS patients?

Various efforts have been made over the years to have a uniform yardstick for judging the quality of life of patients with cardiovascular diseases^20^, with special emphasis on the brevity of questionnaire completion and self-reported perceptions^21–24^.

Regarding the assessment of quality of life (QoL), the health-Related QoL questionnaire has proven to be valid and easy to administer in patients with ischemic heart disease and heart failure^20^. This assesses two different pathways on how the patient copes his disease, the physical one and the emotional one^25^.

Other questionnaires with known psychometric validity and widely used for the assessment of the QoL are the Mc-New Heart Disease Health-related Quality of Life questionnaire and the Short form Health Survey 36 (a generic health survey with 36 questions including physical component and mental component)^18^.

About mental and behavioral disorders screening, the GHQ-12 (General Health Questionnaire) is widely validated for the assessment of psychological disorders in primary health care^23^. For the assessment of possible emotional instability^26,27^, it may be used the Ten item personality inventory^28^ which assesses the five major dimensions of personality (extraversion, agreeableness, conscientiousness, emotional stability, openness to experiences).

For the assessment of coping styles, which impact on patients’ mental outcomes^12^, the Brief-cope^22^ may be helpful, a self-reported questionnaire to measure effective and ineffective ways of responding to a stressful life event.

For the assessment of anxiety, an important predictor of QoL^18^, and depression, we have the Hospital Anxiety and Depression Scale, a questionnaire with 14 items with four response options for each.

**Fig. 1:**
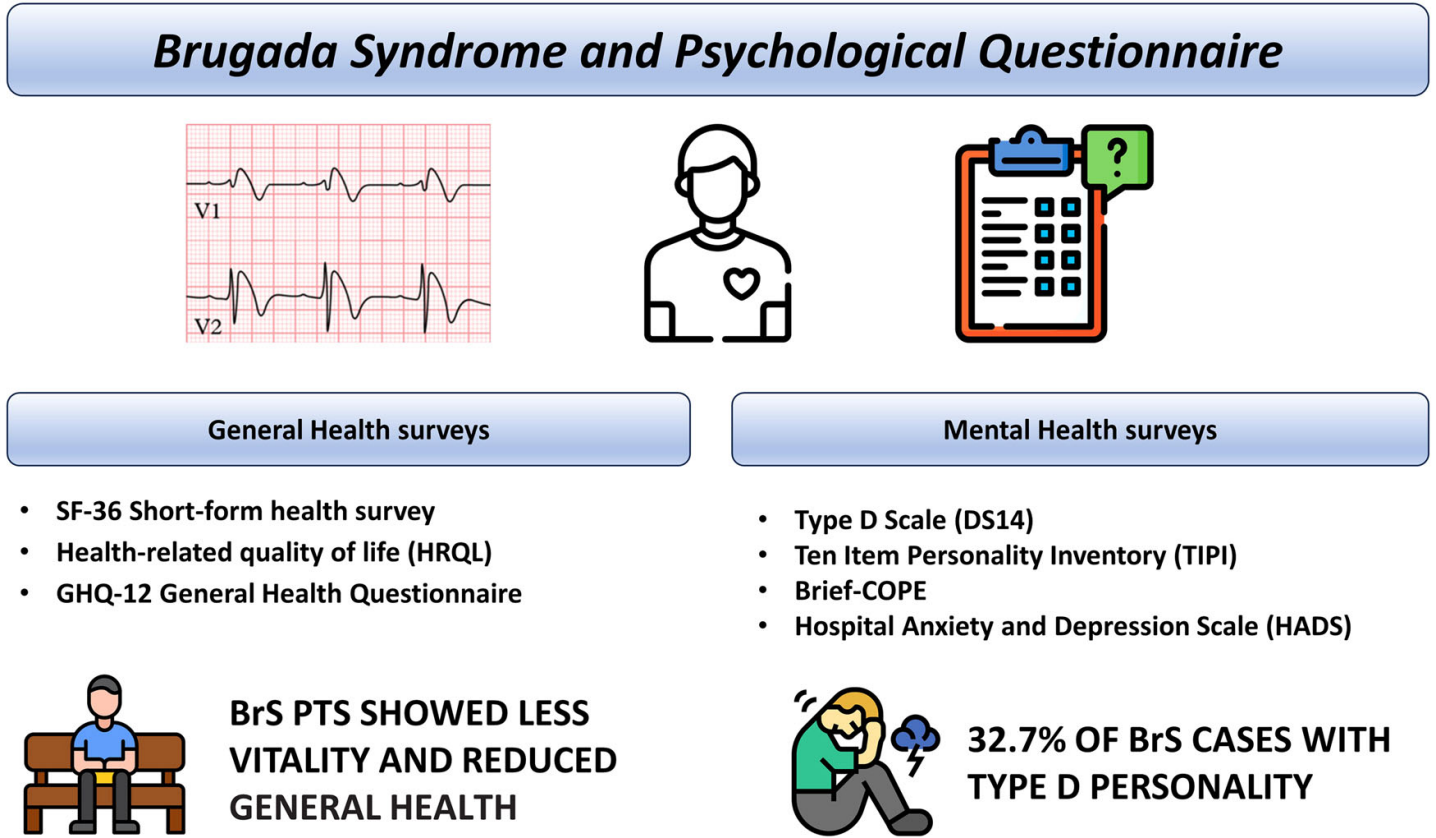
Psychological questionnaires and results provided to the Brugada Syndrome patients. Legend: BrS Brugada Syndrome, PTS patients.

The Oslo Social Support Scale (OSSS-3)^24^ considers the sense of concern perceived by others about the patient’s condition and the type and number of social relationships he or she can rely on. The list of tests evaluated among BrS patients and results comparing BrS patients and controls are provided in Table 2 and Fig. 1.

**Table 2:** Questionnaire evaluating general and mental health status in patients with Brugada syndrome (BrS) and results in comparison with control group

*Study*	year	Num. of patients	Mean age (y)	Male (%)	Questionnaire	Results test in BrS patients compared with controls
Six et al. ^3^	2023	162	49	59%	General Health Questionnaire (GHQ-12)	↑↑
					Oslo Social Support Scale (OSSS-3)	=
					Health-related quality of life (HRQL)	=
					Type D Scale (DS14)	↑↑
					Ten Item Personality Inventory (TIPI)	↓↓
					Brief-COPE	**↑↑**
Sutjaporn et al. ^18^	2022	29	45	96%	Mc-New Heart Disease Health-related	NA
					Quality of Life	↑↑
					Short-form health survey (SF-36)	NA
					Hospital Anxiety and Depression Scale (HADS)	NA
					Grooved Pegboard	NA
					Consortium to Establish a Registry for Alzheimer’s Disease–Neuropsychological battery (CERAD)	NA
Probst et al. ^8^	2011	190	53	79%	Short-form health survey (SF-36)	↑↑↑
						General - mental health (GH – MH) and vitality (VT)
						are lower in the BrS population

### Diagnosis of BrS in patients with mental diseases and use of psychiatric drugs

The impact of BrS diagnosis is heterogenous. Some patients cannot manage well the diagnosis and symptoms of a hereditary heart disease, others may manage better especially if their family members are asymptomatic and do not carry the risk gene^29^.

Jespersen et al. ^19^ found in a cohort of 263 consecutive patients with BrS diagnosed between 2006 and 2018 from a national registry in Denmark, that before BrS diagnosis about 15% of patients had an history of psychiatric disorders.

Patients with BrS and history of psychiatric disorders (40 out of 263) were taking antidepressants (42.5%), anxiolytics (27.5%), and antipsychotics (17.5%).

**Fig. 2:**
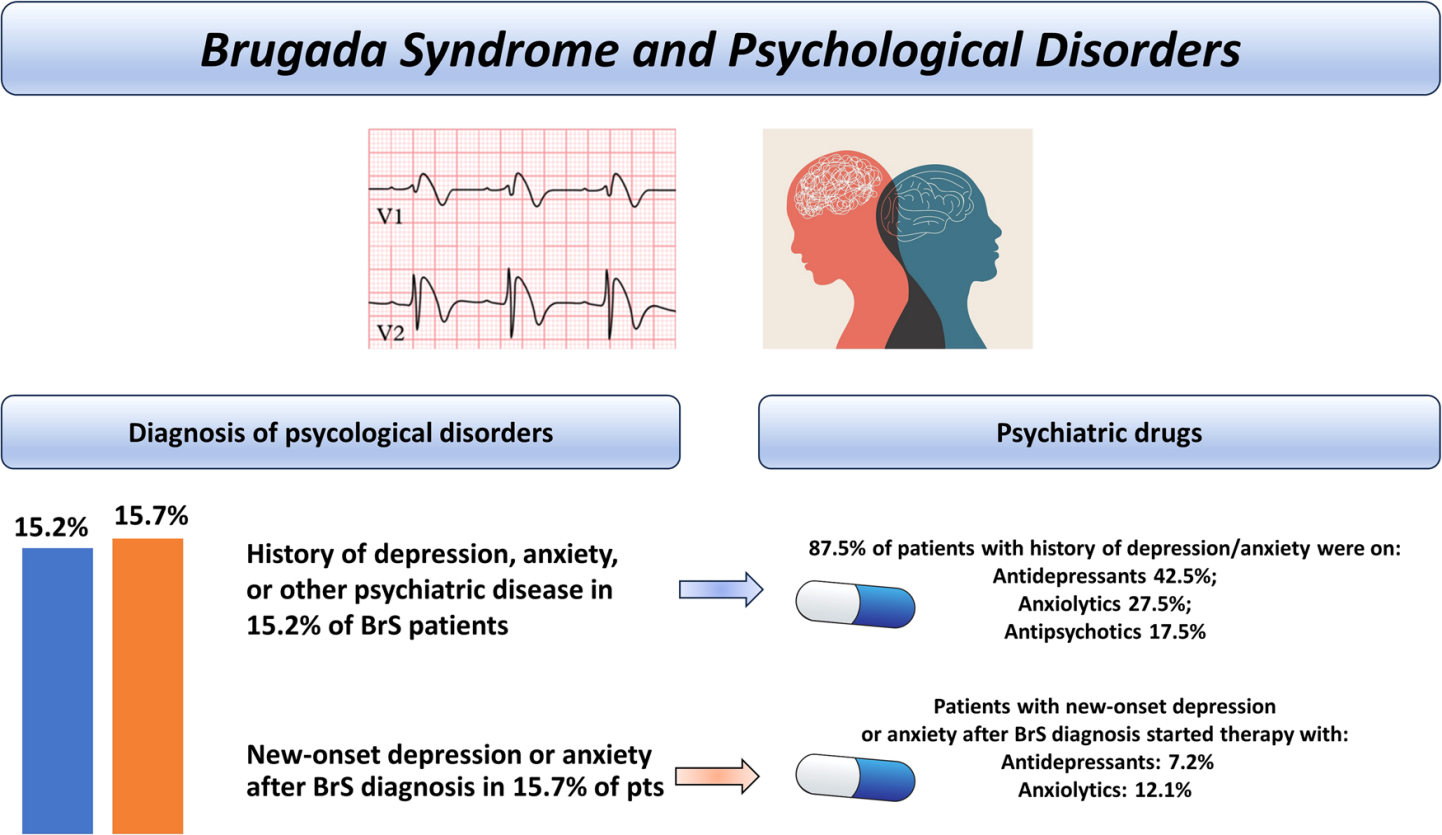
Prevalence of psychological disorders and psychiatric drugs administered among patients with Brugada syndrome before and after its diagnosis Legend: BrS Brugada Syndrome, PTS patients.

On the other side, 35 out of 223 patients (15%) developed anxiety or depression following BrS diagnosis. Some of these patients started therapy with antidepressants (7.2%) or anxiolytics drugs (12.1%) (Fig. 2, Table 3). In the same Danish registry, authors found that several BrS patients were treated with psychiatric drugs not recommended. This tendency was higher among patients with BrS who developed new-onset depression or anxiety (*n* = 18/35, 51.4%)^19^. This finding is line with several studies that have correlated ion channel disease with psychiatric disorders^30,31^.

**Table 3:** Prevalence of drug related to depression or anxiety in patients with Brugada Syndrome, before and after the diagnosis of this syndrome

*Study*	year	Num. of patients	Mean age	Male (%)	Drugs related to depression or anxiety (*n*, %)
Jespersen et al. ^19^	2023	263	46	76%	
*Patients with htx of psychiatric disorders*		40	48.4	60%	Antidepressants: 17 (42.5)
*Patients with no psychiatric disorders before BrS diagnosis*		223	46	72.6%	Anxiolytics 11 (27.5) Antipsychotics: 7 (17.5) Antidepressants: 16 (7.2) Anxiolytics: 27 (12.1)

Many psychiatric drugs are contraindicated in Brugada syndrome (tricyclic antidepressants, mood stabilizers and antipsychotics)^32–39^. Lithium, a well-known drug used for mood disorders, may act as a blocker of the cardiac sodium channels even at concentrations below those achieved in therapy and could unmask BrS^32^. Tricyclic antidepressants, as desipramine and amitriptyline, have been reported to unmask BrS^33,34^. Moreover, also antipsychotics as quetiapine and clozapine may unmask this feature^37–39^.

It is therefore of paramount importance a shared pharmacological evaluation between cardiologist and mental health physicians, in selected cases.

### Potential psychological impact of ICD implant

The implantation of ICDs is a crucial aspect in the history of this syndrome. Despite the undeniable medical benefits of ICD treatment, living with an ICD and underlying heart disease can lead to psychological distress, with 20–30% of patients experiencing significant levels of anxiety and depression^40–42^. Prevalence of anxiety and depression is similar among patients suffering of different inherited channelopathies^40^. However, in a large European survey including 1644 patients with ICD about 75% of the patients had an improved quality of life after device implantation, but nearly 40% had some worries about their device^41^. Van der Broek et al found that, in a longitudinal study evaluating 343 patients and partners following ICD implantation, partners experienced more anxiety and patients more depression^42^.

Probst et al. evaluated a cohort of 190 BrS patients^8^. Authors found that half of the BrS symptomatic implanted patients and a quarter of the asymptomatic implanted were anxious of the potential side effects of the ICD.

Additionally, there was a strong relationship between age and negative impact related to the ICD. Indeed, younger patients referred that ICD had a worst impact on quality of life^43^.

The experience of shock delivered by ICD was also associated with a quality-of-life deterioration: 76% of patients who received at least one shock considered that the ICD was responsible for deterioration in their quality of life, compared with 53% of patients who never received a shock. Moreover, patients who experienced an ICD shock were more frequently concerned about potential complications (58%) than those who never received a shock (32%)^8^.

### Which predisposing factors for new-onset psychological disorders should be considered?

The psychological impact and repercussions of this diagnosis are linked to several patient-related variables, possible comorbidities and coping behaviors, varying widely between patients. In this regard, screening BrS patients for previous psychiatric illnesses is important for a proper follow over time.

Personality plays a significant role in determining chronic stress levels^21^. Two overarching personality traits, negative affectivity (NA) and social inhibition (SI), are particularly relevant in this context. NA refers to a tendency to experience negative emotions consistently across various situations, leading to feelings of dysphoria, anxiety, and irritability. SI, on the other hand, involves inhibiting the expression of emotions or behaviors in social settings to avoid disapproval from others. Individuals with NA and SI are classified as having a distressed or Type D personality due to their susceptibility to chronic distress^21^.

Type D personality is more common in patients with BrS than in the general population. Six et al. in a cohort of 165 BrS patients found a prevalence of type D personality of 37.2%^3^ (Fig. 1). Moreover, Symptomatic disease presentation and older age are significantly associated with new-onset depression or anxiety^19^.

However, the information about potential psychological disorders is a dynamic element and requires serial evaluation^43–47^.

### What is the psychological impact of out-of-hospital cardiac arrest in BrS?

BrS patients are at higher risk of cardiac arrest, as outlined before^7^. Therefore, some of these patients may experience out-of-hospital cardiac arrest (OHCA), usually before the time of BrS diagnosis. However, it is well demonstrated that a not negligible percentage of patients after OHCA may experience psychological distress, such as the development of depression and anxiety, cognitive impairment, and fatigue, which negatively affect the quality of life of both patients and their relatives^47,48^. Moreover, the incidence of depression in this population is higher in younger patients (<50 years old). This is not to be underestimated as having a longer life expectancy than older people it is essential to act by improving their quality of life^49^. The recognition of varying levels of psychological distress over time holds significance as it may impact cardiac outcomes differently among patient groups. Indeed, distinct subtypes of depression may influence behaviors related to secondary prevention rather than directly affecting cardiac outcomes^21^. Furthermore, depression could be one of several factors potentially responsible for the increased mortality of post-arrest patients compared to the healthy reference population^50^. This psychological issue is often underestimated and not sufficiently investigated and managed in the months following cardiac arrest and this is even more striking in BrS patients, considering both the fact that they are often young patients and that they are already at greater risk of psychological distress as outlined before. For this reason, as suggested by the European guidelines on the treatment of OHCA patients, a systematic follow-up of all cardiac arrest survivors, including BrS patients, after hospital discharge is needed^51^.

### Future perspectives and potential intervention

Many patients with hereditary heart disease report levels of psychological distress that suggest the need for clinical intervention and report a reduced health-related quality of life compared to the general population^52–55^.

Psychological interventions can optimize patients’ treatment expectations, leading to improvements in mental quality of life^56^. Psychological interventions such as cognitive behavioral therapy can effectively modify personality traits, with effects persisting over time. The optimal duration of such interventions appears to be between 4 and 8 weeks, with no additional benefits observed beyond 8 weeks.

For this reason, screening BrS patients at diagnosis for the above-mentioned conditions by administering questionnaires could be useful to tailor any psychological/psychiatric follow-up to the individual’s needs, as well as to optimize therapy compliance.

In the management of Brugada syndrome, it is crucial to adopt a multidisciplinary approach addressing both psychological and pharmacological aspects. Additionally, patients with BrS who develop new-onset anxiety or depression are often prescribed medications that should be avoided in their condition^19^. Targeted psychological interventions for patients and their families, such as counseling, cognitive-behavioral therapy, and peer support groups, can play a crucial role in promoting the emotional well-being and psychological resilience of patients with this complex cardiac condition.

Psychological/psychiatric counseling should not stop at the stage of diagnosis but continue as needed. This should aim not only at alleviating psychological distress, but also at developing or consolidating coping-responses the patient may benefit from^12^.

### Limitations

BrS is a rare disease, therefore few data are available in literature and it is not possible to provide strong recommendations on the attitude to take towards this category of patients. Moreover, there is a lack of information in the pediatric population.

This review does not deal in detail with the psychological and attitudinal therapies to be employed for each patient category, as they are beyond the scope of this text.

### Summary of evidence

- Previous history of psychiatric disorders before BrS diagnosis was found in 15% of patients in a registry of 263 BrS patients^19^;
- Prevalence of Type D personality is 32.7% in a registry of 162 BrS patients^3^;
- After BrS diagnosis, patients can develop anxiety or depression, the incidence is higher among symptomatic patients (19.3% vs 13%)^19^;
- Anxiety on health status is common in BrS patients about 49% can develop it (41% moderate, 8% severe)^8^.

## Conclusions

The diagnosis of Brugada syndrome can lead to psychological and mental health repercussions in several ways. Screening newly diagnosed BrS patients with self-reported and brief questionnaires could help identify those who may require referral, to a mental health specialist. A multidisciplinary approach including cardiologists and psychiatrists in order to establish the most suitable psychopharmacological treatment should be considered.

## Data Availability

No datasets were generated or analyzed during the current study.
